# Impact of career choice motivation on academic burnout in senior dental students: A cross-sectional study

**DOI:** 10.1186/s12909-020-02475-w

**Published:** 2021-01-14

**Authors:** Simin Z. Mohebbi, Mahdia Gholami, Mostafa Chegini, Younes Ghoreyshi, Ronald C. Gorter, Hoda Bahramian

**Affiliations:** 1 Research Center for Caries Prevention, Dentistry Research Institute, Tehran University of Medical Sciences, Tehran, Iran; 2 Department of Community Oral Health, School of Dentistry, Tehran University of Medical Sciences, Tehran, Iran; 3 Department of Dental Educational Research / Oral Radiology, Academic Centre for Dentistry Amsterdam (ACTA), Amsterdam, The Netherlands

**Keywords:** Academic Burnout, Career Choice Motivation, Burnout Clinical Subtype Questionnaire, Dental Students

## Abstract

**Background:**

Motivation triggers all human activities including learning and lack of career motivation can lead to decreased efficiency. This study assessed the association between academic burnout and career choice motivation in senior dental students.

**Methods:**

This cross-sectional study was performed on senior dental students of all four dental schools in Tehran in 2017. Dental students voluntarily filled out a 33-item questionnaire that comprised three sections. The first section included the Burnout Clinical Subtype Questionnaire (BCSQ-SS) with 12 questions addressing academic burnout. The second section consisted of 8 questions about career choice motivation, and the third section concentrated on 13 questions dealing with demographics. The individual scores of each section and the total scores were reported. The factor analysis of 8 questions about dental career choice motivation yielded 3 factors of social status and security, altruism, and others’ advice motivations. The data were analyzed using regression test.

**Results:**

Totally, 131 students filled out the questionnaire (response rate = 86%). The total score of academic burnout was 38.89% ± 1.13%. The highest and the lowest burnout scores belonged to the domains of “overload” (46.69%± 1.46%) and “neglect” (31.98%± 1.32%), respectively. The most and the least frequently mentioned source of motivation for choosing dentistry were high income and failing to enter other fields, respectively. The burnout score was higher in students with altruism motivations (*P* = 0.007) and lower in students with others’ advice motivations (*P* = 0.004). The burnout score was higher in students with inadequate or moderate financial support from the family and lower in students whose mothers’ educational level was high-school diploma or lower.

**Conclusion:**

Senior dental students in Tehran encountered different levels of academic burnout. In the present study, low financial support and altruism as career choice motivations were associated with higher level of academic burnout.

## Background

Occupational burnout refers to decreasing coping ability of an individual due to stressors and subsequent physical and emotional burnout, which would lead to a negative self-impression, negative attitude towards the occupation, and feeling of getting disconnected from others [1].

Academic burnout is relatively common among students and refers to a sense of fatigue due to high academic demands and requirements (educational burnout), negative attitudes and lack of interest in the field of study (academic disinterest), and sense of incompetence in education (academic inefficacy) [2]. Academic burnout is often associated with sadness and loss of interest which can decrease motivation and negatively affect the quality of learning and performance [3], especially in dental students due to their heavy educational curriculum [4–6]. Thus, the screening of dental students for academic burnout is imperative to come up with strategies for its prevention and subsequent adverse effects on their physical and mental health.

The prevalence of academic burnout differs worldwide [7–9]. It often occurs as the result of inability to cope with the educational curriculum [10–12]. Studies reveal that regardless of the current educational system, the rate of academic burnout is high among dental and medical students [6, 10, 13, 14]. Academic burnout can result in quitting, missing classes, or having a bad mood. It can also cause physical fatigue, insomnia, alcoholism, substance abuse, and family issues [11, 15].

Motivation is an internal force that accounts for the efforts an individual makes at work. There are two main theories, namely content theories and process theories which clarify the nature of motivation, where the former focuses on individual needs that affect behavior in the workplace, whereas the latter addresses equity and expectancy theory [16]. While some researchers contend that being motivated does not necessarily result in positive educational outcomes [17], others believe that internal motivation facilitates deep learning, better academic performance, and positive student well-being [18]. People have different motivations for their activities, and their priorities are not the same [19–21]. Individuals’ career choice motivation may not be the same either. Lack of career motivation can lead to decreased efficiency [21]. Motivation triggers all human activities including learning. It empowers the learners and guides their activities [20]. The presence of motivation in the process of education enhances learning, facilitates communication, decreases anxiety, and results in creativity in learning. People have widely variable motivations for learning and selecting their careers [22]. Factors commonly considered as career motivations include wages and benefits, vibrant working environment, occupational safety, interest in the occupation and its related social communications, the nature of occupation, social position and appreciation, and attractiveness of the job [23]. Career choice motivations often depend on the family and educational environments, influential people with diverse personal, social, cultural and financial backgrounds, interests and values, beliefs, personal characteristics and personality traits as well as capabilities and skills of an individual. The literature shows that gender, race, and level of parents’ education can also impact the individuals’ career choice motivations [24]. Comparing motivational factors in students of Medicine and Dentistry, we found that dental students are more motivated by status, security, and the nature of dentistry (e.g. regular working hours, self-employment, independence, and financial gain). However, medical students are motivated by factors related to career opportunities, patient care, working with people, use of personal skills, and interest in science [25].

Physicians and dental clinicians often tolerate high levels of stress and 21% of Dutch dentists have certain risks for burnout [26]. Around 13% of dental clinicians experience high levels of occupational burnout [27]. Also, evidence shows that dental clinicians are under much higher tension and stress due to the nature of their occupation compared with other jobs and reported existence of variable burnout scores among dental students [4–6, 28]. Previous studies on dental students have shown some stress factors, such as lack of leisure time, examination anxiety, the transition stress in adapting themselves to the demands of the clinical phase of dental education, patient-related stress, study obligation ,and pressure [5, 6].

The association between academic burnout and career choice motivation is shown in some studies. Pagnin et al. have reported medical students motivated for their future career by personal illness or family member's illness or death to be at a great risk for burnout in comparison to the students with other motivations [14]. Karaoglu & Seker in 2010 found that medical students who are under external pressure to become a doctor and their expectation from medical career is reaching to a better economic condition are experiencing more anxiety and depression that can be preceded by burnout [29]. Never the less, such associations have been rarely investigated about dental students. In this study we aimed to assess the relationship between academic burnout and career choice motivations in senior dental students.

## Methods

This cross-sectional study was performed on senior dental students of all four dental schools in Tehran (Tehran University of Medical Sciences, Shahid Beheshti University, Shahed University, and Islamic Azad University of Medical Sciences) in 2017. Since the regression models were used for data analysis, 8–10 samples were selected for each independent variable according to the rule of thumb [30] and the minimum sample size was calculated to be 100 dental students. As the total number of senior dental students studying in the four major dental schools in Tehran exceeded our minimum sample size (*n* = 230), 152 dental students were selected using simple random sampling from the lists of the students who had announced their consent to attend the study. The participants were reached in one of their morning theoretical classes at the beginning of the final semester; therefore, the weekly schedule of senior students' courses was checked. We had asked for the professors’ permission before elaborating on our research objectives. The questionnaires were distributed and after 10 minutes, the questionnaires were collected.

The data were collected using a 33-item questionnaire in 3 sections. The first part included 12 items extracted from the Iranian version of Burnout Clinical Subtype Questionnaire (BCSQ-12-SS) to address three domains of overload, lack of development, and neglect. The validity and reliability of this questionnaire, i.e.“BCSQ-12-SS” [31] had been previously confirmed in the Iranian population [28]. The students expressed their level of agreement with the statements discussed in this questionnaire using a 5-point Likert-scale scoring system ranging from (1) “I totally disagree” to (5) “I totally agree”. The minimum and maximum burnout scores that could be acquired were 12 and 60, respectively. In this study, the burnout scores were reported as percentages (from 20–100%). The separate scores of the three domains of overload, lack of development, and neglect were also standardized by calculating from 100.

The second part of the questionnaire included 8 questions regarding career choice motivations [32], and dental students were allowed to select more than one choice (Table 1).

**Table 1:** Questions regarding career choice motivations

Question Number	Question
1	Failure to enter other educational fields
2	Advice by parents or others
3	Interest in dentistry as a combination of art and science
4	High level of income
5	Occupational independence
6	Good social position
7	Helping others to regain their health or improve their appearance
8	Being easier than medicine

The third part of the questionnaire included 13 questions about the demographic information of students including gender, marital status, father’s level of education, mother’s level of education, and financial support by the family, city of residence, economic status of the family, Grade Point Average (GPA) of the last semester, and so on [28].

### Statistical analysis

Data were analyzed using SPSS version 21 (SPSS Inc., IL, USA). To identify the underlying dimensions for career choice motivations, an Exploratory Factor Analysis (EFA)‎ with principal component method and varimax rotation was applied. Each item that loaded at 0.60 or greater on only one factor was included as an item for a given factor. Based on the factor analyses, new variables were formed by summing up the values of the original variables. Linear regression analysis was employed for the purpose of evaluating the association between educational burnout and career choice motivation while controlling the students' background as probable confounders. Weighting was performed prior to inferential analysis. To this end, the number of senior dental students in each dental schools of Tehran was divided by the total number of participants.

## Results

Of 152 dental students, 131 completely filled out and returned the questionnaire (response rate = 86%) The questionnaires with missing data were omitted from the study. The mean age of the participants was 24.4 years and 48.1% were males (Table 2).

**Table 2:** Demographic information of participants (*n* = 131)

Variable	Categories	Number	Percentage
**Gender**	**Male**	63	48.1
	**Female**	68	51.9
**Marital status**	**Single**	108	82.4
	**Married**	23	17.6
**Father’s educational level**	**Illiterate**	1	0.8
	**Elementary-middle school**	2	1.5
	**High school-high school diploma**	19	14.5
	**College-bachelor’s degree**	73	55.7
	**Master’s degree or higher**	36	27.5
**Mother’s educational level**	**Illiterate**	1	0.8
	**Elementary-middle school**	7	5.4
	**High school-high school diploma**	32	24.4
	**College-bachelor’s degree**	70	53.4
	**Master’s degree or higher**	21	16
**Financial support by the family**	**Inadequate**	9	6.9
	**Moderate**	83	63.3
	**Good**	39	29.8

The students’ mean grade point average (GPA) was 15.94. Table 3 presents the frequency of students’ responses (weighted) to the questions of different domains of BCSQ-SS. Regarding the first question of overload domain “I think the dedication I invest in my education is more than what I should for my health”, In general, 40.1% had chosen the “I agree” or “I totally agree” choices. Regarding the second question of overload domain “I neglect my personal life when I pursue important achievements in my education”, the most frequent choice was “I disagree” with 37.8% frequency. Regarding the third question of overload domain “I risk my health when I pursue good results in my education”, the most frequent choice was “I disagree” with 41.9% frequency. And the fourth question of overload domain “I overlook my own needs to fulfill educational demands”, “I agree” was the most frequent answer choice with 36.8% frequency.

**Table 3:** Frequency of students’ responses to different domain of BCSQ-SS (%)

	Totally disagree	Disagree	Not sure	Agree	Totally agree
**Dimensions of overload**					
1. I think the dedication I invest in my education is more than what I should for my health	10.2	27.5	22.2	29.5	10.6
2. I neglect my personal life when I pursue important achievements in my education Frequency	11.8	37.8	16.3	28.2	5.9
3. I risk my health when I pursue good results in my education Frequency	12	41.9	15.3	28.4	2.3
4. I overlook my own needs to fulfill educational demands Frequency	9.1	30.9	17.7	36.8	5.4
**Dimensions of lack of development**					
5. I would like to study in another field that is more challenging for my abilities	24.7	31.7	15.9	19.4	8.4
6. I feel that my educational field is an obstacle to the development of my abilities	28.2	31.3	13.0	21.3	6.2
7. I would like to study in another field where I can better develop my talents	25.0	35.4	14.0	19.5	6.0
8. My field of education does not offer me opportunities to develop my abilities	18.5	32.3	22.3	23.9	3.0
**Dimensions of lack of development**					
9. When my grades do not turn out as they should, I stop trying	23.6	36.8	19.8	17.0	2.8
10. I give up in response to difficulties in my education	26.1	38.0	21.4	10.9	3.7
11. I give up in the face of any difficulties in my educational tasks as a student	25.4	40.8	21.5	10.0	2.3
12. When the effort I invest in my education is not enough, I give in	26.1	41.8	19.3	9.8	3.0

Regarding the lack of development domain and the neglect domain of BCSQ-SS, “I disagree” was the most common choice in these domains. (Table 3)

The mean total score of burnout was 38.89%± 1.13%. The mean score of overload, lack of development, and neglect was 46.69%± 1.46%, 37.99%± 1.37% and 31.98%± 1.32%, respectively.

For dental career choice motivations, the factors with their components and their coefficients from EFA (in the parenthesis) are as follows:

Social status and security motivations consisted of good social position (0.8), work independence (0.6), failure to enter other fields of education (-0.6), and high income (0.6).

Altruism motivations included intellectual challenges the students dealt with to help others regain their health or improve their appearance (0.7), and the key characteristics of Dentistry as a combination of art and science (0.8).

Others’ advice motivations included parents’ or others’ advice to choose Dentistry as a career (0.8) and studying Dentistry is easier than Medicine (0.6).

The dental students’ responses regarding their career choice motivations showed that high level of income (90%), good social position (89.9%), and work independence (89.5%) were the most common dental career choice motivations, while failure to enter other fields of education (13.7%) was the least common motivation. (Table 4)

**Table 4:** Dental students’ responses regarding their career choice motivations (*n*=131)

Question		Frequency %	95% CI	
			Lower bound	Upper bound
**1.Failure to enter other educational fields**	Yes	13.7	8.7	20.9
	No	86.3	79.1	91.3
**2.Advice by parents or others**	Yes	54.4	45.9	62.9
	No	45.6	37.1	54.1
**3.Interest in dentistry as a combination of art and science**	Yes	62.2	58.4	74.9
	No	38.8	25.1	41.6
**4.High level of income**	Yes	90.0	83.4	94.1
	No	10.0	5.9	16.6
**5.Occupational independence**	Yes	89.5	82.9	93.8
	No	10.5	6.2	17.1
**6.Good social position**	Yes	89.9	83.2	94.1
	No	10.1	5.9	16.8
**7.Helping others to regain their health or improve their appearance**	Yes	70.8	62.4	78.0
	No	29.2	22	37.6
**8. Being easier than medicine**	Yes	73.6	65.2	80.5
	No	26.4	19.5	34.8

The 8 questions of dental career choice motivations were analyzed by factor analysis, and 3 factors, namely social status and security, altruism, and others’ advice motivations were extracted. The burnout score was found to be significantly higher in students who had chosen dentistry as their field of education due to altruism motivations (*P* = 0.007). Students who had chosen dentistry as their field of education due to others’ advice motivations had a significantly lower burnout score (*P* = 0.004).

Regression analysis of the association between demographic factors and career choice ‎motivations with the burnout score revealed a significant correlation between financial support by the family and academic burnout. It showed that both inadequate (*P* = 0.021, *r* = 12.82) and moderate financial support (*P* = 0.010, *r* = 6.86) increase the burnout score. Also, mother’s level of education equal to or below high-school diploma has a significant correlation with academic burnout and decreases it (*P* = 0.026, *r*=-8.87). Altruism motivations significantly increase the burnout score (*P* = 0.021, *r* = 2.79), while others’ advice motivations significantly decrease the burnout score (*P* = 0.001, *r*=-3.75).

## Discussion

This study assessed the relationship between academic burnout and career choice motivations in senior dental students in Tehran. The total burnout score was found to be 38.89%. The domain scores were 46.69% for overload, 37.99% for lack of development, and 31.98% for neglect. Mafla et al. [33] assessed the prevalence of academic burnout and its association with different factors in dental students in Colombia. They reported that only 7% of dental students met the criteria for academic burnout. Mohebbi et al. [28] assessed the prevalence of academic burnout among Iranian dental students using the BCSQ-12-SS questionnaire. They reported that the average burnout score was 29.6 out of a maximum score of 60. Alemany Martínez et al. [10] assessed the prevalence of academic burnout in postgraduate students of Oral Surgery and Implant, Orthodontics, and Comprehensive Dentistry at Barcelona University. They reported that 2–3% of dental students had high levels of academic burnout. Montero-Marin et al. [31] used BCSQ-SS to assess academic burnout in Huesca University in Spain. They showed that students had high level of academic burnout due to overload, lack of development, and neglect domains, respectively. The prevalence of academic burnout was noticeable in dental students at Santiago University. The findings of the aforementioned study were in agreement with ours because we similarly represented that burnout is common among Iranian dental students. The participants above the 75th percentile of the BCSQ-12-SS score are considered as “high scorers”, whereas those with scores below the 75th percentile are assumed to have some degrees of burnout [31, 34]. Difference in the prevalence of academic burnout values reported in different studies may be due to the use of different data collection tools as well as different environments. However, the high rate of academic burnout observed in dental students in Tehran is alarming which calls for taking necessary measures.

High level of income, good social position, and work independence were the most common career choice motivations of dental students in our study. Failing to enter other fields of education was the least common motivation. In agreement with the study of Hashemipour et al.[35], we showed that the most important motivation for choosing dentistry was relationship with people and income. Khami et al. [32] reported that in dental students with at least one parent being a dentist, the characteristics of this profession had the least effect on those choosing this field of study. Pagnin et al. [14] evaluated medical students and showed that professional autonomy, intellectual curiosity, altruism, and interest in human relationships were the most frequent reasons for choosing medicine. However, medical students motivated by illness or death of a family member had significantly greater emotional exhaustion than those with other motivations.

In our study, GPA score had no significant correlation with academic burnout, which was in agreement with the findings of Maslach [1]. The correlation between burnout and age was not significant in our study. In a study by Krokter Kogoj et al. [13], older dental students showed higher rate of burnout, while Galán et al. [36] reported that younger dental students had higher rate of burnout. In our study, academic burnout had no significant correlation with gender, marital status, university, or city of residence, which was in agreement with the results of Singh et al. [37]. Montero-Marin et al., [31], and Galán et al. [36]. However, Mafla et al. [33]illustrated that marital status had a significant correlation with burnout, which was different from our findings.

The classification of motivations into three categories of altruism, social status and security, and others’ advice motivation revealed lower level of burnout in students with others’ advice motivations and higher level of burnout in students with altruism motivations. This finding indicates that dental students who chose Dentistry based on the advice of their parents or others or because they thought that Dentistry would be easier than medicine, were more satisfied with their conditions. On the other hand, dental students who chose Dentistry since they thought it is a combination of art and science or because they wanted to resolve the health issues or esthetic needs of patients were not satisfied with their conditions and their expectations had not been met. Higher altruism motivation has been shown to support medical students against high educational burnout [38]. Higher burnout levels in those with altruism motivations in the present study may reveal that such students are not meeting what they had expected in the field. More qualitative in-depth studies are needed to delve into the underlying factors in curriculum design and/or implementation. Moreover, the career choice motivation questions of the current study could have ‎benefited from Likert scale response alternatives instead of a binary “yes /no” to reveal the range of the ‎importance and priority.

In our study, mother’s level of education had a significant correlation with burnout in students, and the level of burnout was lower in dental students whose mothers held high-school diploma or lower-level certificate. This finding indicates that although mothers with lower level of education probably encouraged their children to pursue dentistry and reach high academic degrees more, it seems that more educated mothers in Iran are more stressful and preoccupied with their children’s future which causes stress and burnout. In Iran, the same as some other countries, compared to fathers, the effect of mother’s education on their children’s health and achievement is more dominant. Consequently, it is worth investing more time and effort in mothers' education. [39, 40] Financial support by the family also had a significant correlation with burnout. Two assumptions are considered by the authors to explain this finding. First, in the presence of good financial support from the family, the student have no financial concerns and better focus on studying. This could decrease burnout. However, in the absence of sufficient family support, the burnout increases. The other assumption speculates that higher financial support by the family brings about higher convenience, which may decrease the students’ performance efficiency and subsequently their motivation. Our findings were somehow in favor of the first assumption. Regression analysis revealed higher rate of burnout in dental students with inadequate financial support from the family and those with altruism motivations. In addition, lower rate of burnout existed in the students whose mothers’ educational level was equal to or below high-school diploma and had others’ advice motivations.

Our study highlighted the challenges that students were engaged with causing them experience more ‎burnout such as low financial support from family and their career choice motivations as altruism ‎that was among their expectations that had not been met in field of dentistry. Thus, it is suggested to serve some strategies and mentorship to deal with such situations to prevent or decrease future probable burnout.

This study was conducted in Tehran and on senior dental students. Being the final outcome from the dental school, we focused on senior dental students as the burn out and its determinants ‎may change throughout the study period. Thus, the obtained results may have limitation in generalizability for the other parts of the country or for other countries. Further studies are also required to find the reasons for high level of burnout in dental students in Tehran and come up with strategies to decrease it. We have included motivation in this study which may itself be related to competence, autonomy, and relatedness [41, 42]. In future studies, it is suggested to investigate each of them separately for dental students. Furthermore, it is suggested that studies based on self-determination theory would be designed and conducted to elaborate on the subject more precisely.

## Conclusion

The rate of academic burnout was relatively high in senior dental students attending dental schools in Tehran. Lower financial support and career choice motivations such as altruism motivations were associated with burnout which highlights the challenges such students encountered to fulfil their professional activities.

## Data Availability

The datasets used and/or analyzed during the current study are available from the corresponding author on reasonable request.

## Abbreviations

BCSQ-12-SS: Burnout Clinical Subtype Questionnaire
GPA: Grade Point Average
EFA: Exploratory Factor Analysis
